# Chemopreventive Effects of Propolis in the MNU-Induced Rat Mammary Tumor Model

**DOI:** 10.1155/2020/4014838

**Published:** 2020-02-26

**Authors:** A. F. Gal, L. Stan, F. Tăbăran, D. Rugină, A. F. Cătoi, S. Andrei

**Affiliations:** ^1^Faculty of Veterinary Medicine, University of Agricultural Sciences and Veterinary Medicine Cluj-Napoca, 3-5 Mănăștur Street, 400372 Cluj-Napoca, Romania; ^2^Faculty of Food Science and Technology, University of Agricultural Sciences and Veterinary Medicine Cluj-Napoca, 3-5 Mănăștur Street, 400372 Cluj-Napoca, Romania; ^3^Faculty of Medicine, “Iuliu Haţieganu” University of Medicine and Pharmacy, 8 Victor Babes Street, 400000 Cluj-Napoca, Romania

## Abstract

Currently, one of the central problems in cancer management is the relapse of disease following conventional treatments, yet few therapeutic agents targeting resistance and tolerance exist. Propolis is known as a healing agent since ancient times. Therefore, over time, its curative properties have kept the interest of scientists, thus leading permanently to investigations of its other possible undiscovered effects. In this context, current experiments were performed to establish the chemopreventive potential of propolis extract (PE) (1.05 mg/kg BW/day) in N-methyl-N-nitrosourea- (MNU-) induced rat mammary tumors. MNU-inoculated/PE-treated rats had tumors of different physical attributes compared with control rats MNU-inoculated. The number of developed tumors (mean 49% versus 100%), incidence (mean 49% versus 100%), multiplicity (1.8 versus 3.7 (*p* < 0.001)), tumor volume (mean 10 cm^3^ versus 16 cm^3^ (*p* < 0.001)), and weight of the tumor mass (mean 7.42 g versus 9.00 g (*p* < 0.05)) were noted. The numbers of grade I tumors recorded for MNU-inoculated rats were 24 (Group 1) and 7 (Group 2) for MNU-induced/PE-treated rats. In the serum of rats MNU-inoculated/PE-treated were found higher levels of antioxidative enzymes (SOD, CAT, and GPx) than in MNU-induced. Taken together, these data indicate that propolis could be a chemopreventive agent against MNU-induced mammary carcinogenesis.

## 1. Introduction

The human breast cancer and the canine mammary cancer are the most frequently detected malignancies in women and female dogs worldwide, in which, regardless of an intensive cancer control effort, it remains one of the main leading causes of cancer deaths [1, 2]. Nowadays, a lot of efforts are made in order to find new complementary and alternative therapies for different cancer types, because of their highly resistance or tolerance to the conventional treatment. The curative properties of the bee glue called propolis have kept the interest of scientists worldwide, leading permanently to investigations of its other undiscovered possible effects. Till now, propolis received the scientific attention due to its proved antioxidant [3–6], anti-inflammatory [7, 8], and antitumor [3, 9–11] properties. Propolis is generally composed of 50% to 60% resins and vegetable balsam, 30% to 40% bee wax, 5% to 10% essential and aromatic oils, 5% pollen grains, and 5% other substances as micronutrients or small amounts of vitamins B1, B2, B6, C, and E [12]. In these 5% of other substances, more than 300 bioactive molecules are present, between them being phenolic acids, flavonoids, diterpenoids, and triterpenoids [13]. The European propolis contains predominantly phenolic compounds, including several flavonoids [14]. The chemical composition of propolis could be influenced by the geographical region and bee species which collect the raw material and produces it. Consequently, biological properties of propolis from different geographical regions could exert particular biological properties.

The literature data contain *in vitro* studies about many propolis samples resulted from various geographic locations, which have been investigated for their antitumoral activities [7, 10, 15–17]. For instance, propolis originally from Chile proved to have an antiproliferative capacity on KB (human mouth epidermoid carcinoma cells), respective on Caco-2 (colon adenocarcinoma cells) and DU-145 (androgen-insensitive prostate cancer cells) human tumor cell lines [10]. There are several studies done on mammary tumor cells, in which a major compound found in propolis such as caffeic acid phenethyl ester inhibited MCF-7 (hormone receptor positive, HR+) and MDA-231 (a model of triple-negative breast cancer) tumor growth, both *in vitro* and *in vivo* without affecting the normal mammary cells [18]. The same compound was able to decrease the malignancy potential in breast cancer stem cells, by inhibition of self-renewal, progenitor formation, clonal growth in soft agar, and concurrent significant decrease in CD44 content [19]. A recent paper sustains that caffeic acid phenethyl ester is rather most efficient than caffeic acid, inducing cell cycle arrest in S phase and triggering apoptosis in the triple-negative human Caucasian breast adenocarcinoma line cells (MDA-MB-231) [20]. In a similar report, caffeic acid phenethyl ester inhibited breast cancer MDA-MB-231 cells proliferation, activating apoptosis and autophagy, and inhibiting TLR4 signaling pathway [21]. The antiproliferative and proapoptotic activity of Lebanese propolis was demonstrated on breast adenocarcinoma MDA-MB-231 cells, Jurkat leukemic T-cells and glioblastoma U251 cells [16]. Therefore, more papers sustain that the apoptosis is the mechanism involved in the death of tumor cells as human lung adenocarcinoma epithelial (A549), human cervical adenocarcinoma (HeLa), and human breast adenocarcinoma (MCF-7) treated with propolis [22–24].

In literature, several *in vivo* studies assessing the properties of propolis in carcinogenesis were reported, but particularly the clinical trials are scarce. Usually, in experimental studies of breast cancer, the main animal species used are rats or mice, due to the high similarity between human and rodent's mammary, thus being possible to foresee the development process of mammary carcinogenesis in both species [25]. The common carcinogenic agent used in rodents to induce breast cancer development is N-methyl-N-nitrosourea (MNU) [26]. The most malignant lesions induced were carcinomas of the cribriform and papillary types [27].

In this context, this study came to prove that propolis could be a chemopreventive agent against MNU-induced mammary carcinogenesis. To achieve this goal, one propolis sample collected from Transylvania region of Romania was examined for its chemopreventive potential of a long-term day-to-day administration of propolis in MNU-induced rat mammary tumors. Consequently, the data resulted contributes to a better understanding of the geographical region influence to the composition and biological properties of propolis related to those reported in other studies from other parts of the world.

## 2. Materials and Methods

### 2.1. Animals

Thirty-days-old juvenile female Sprague–Dawley rats acquired from the Cantacuzino Institute, Romania, were utilized. The rats were acclimated to laboratory environments in advance of the study, being kept under standard conditions (22–23°C, humidity 60%, light-dark cycle 12 h). All experiments were conducted in agreement with the European Union regulation on animal testing and performed according to the practices of the Romanian Board of Animal Research, having permission of Committee of Animal Ethics of the University of Agricultural Sciences and Veterinary Medicine of Cluj-Napoca (UASVMCN), Romania (UASVM Bio-Ethical Committee Agreement Form no. 9753/22.06.2016).

### 2.2. Preparation of Propolis Extract (PE)

Propolis was collected from the Corund area, Transylvania, Romania (46° 28′ 13^″^ N 25° 11′ 8^″^ E). Briefly, preparation of propolis extract (PE) used in this experiment was done accordingly: 1 g propolis was left to macerate overnight in 30 mL of 95% ethanol at room temperature and under continuous agitation. Then, the extract obtained was filtered and the residue was extracted again under the same conditions. Finally, both extracts were mixed up to a final volume of 100 mL adjusted with ethanol. PE was analyzed for (a) total flavones and flavonols content by a method based on aluminum chloride complex formation (equation of calibration curve for methanolic galangin (4–30 *μ*g/mL) *Y* = 2.04832 × *X* − 0.00233; *r*^2^ = 0.99935), (b) total flavanone and dihydroflavonol content through the colorimetric method with dinitrophenylhydrazine (equation of calibration curve of methanolic pinocembrin (0.2–2.0 mg/mL) *Y* = 0.11034 × *X* − 0.00416; *r*^2^ = 0.99910), and (c) total phenolics by the Folin-Ciocalteu method (equation of calibration curve *Y* = 0.00709 × *X* − 0.00109; *r*^2^ = 0.99932 of methanolic mixture pinocembrin and galangin at a 2:1 ratio (w/w) in the concentration range of 25–300 *μ*g/mL) according to methods previously published [28, 29].

### 2.3. Experimental Design

MNU was utilized to induce mammary tumors (Sigma-Aldrich Chemical Co., St. Louis, USA). MNU was dissolved in standard saline solution, being utilized immediately after preparation. The experimental model of induced carcinogenesis was previously used in our laboratory [30]. The female rats were divided in four groups (10 rats per group) and treated as follows: (a) Group 1, inoculated with a single dose of 55 mg MNU/kg body weight (BW) intraperitoneally (i.p.) and received regular rat food (provided by Cantacuzino Institute, Bucharest, Romania); (b) Group 2, inoculated with a single dose of 55 mg MNU/kg BW i.p. and received regular rat food supplemented with PE in a dose of 1.05 mg/kg BW/day (i.e., 15 droplets of PE/kg BW/day, which were applied on the pelleted diet to avoid the daily handling stress of the rat individuals in this group); (c) Group 3, inoculated with a single dose of saline solution i.p. and received regular rat food supplemented with PE in a dose of 1.05 mg PE/kg BW/day (i.e., 15 droplets of PE/kg BW/day, which were applied on the pelleted diet to avoid the daily handling stress of the rat individuals in this group); (d) Group 4, inoculated with a single dose of saline solution i.p. and received regular rat food. In our experimental model, the daily dose of PE supplemented in the diet of rats was 1.05 mg/kg BW/day.

PE administration was introduced in the diet at the age of 33 days (i.e., 4 days before MNU administration) in order to ensure a proper body condition before the inoculation of the carcinogenic agent. Rats from Groups 2 and 3 were housed alone to ensure the ingestion of the whole daily dose of PE. Rats from Groups 1 and 4 were kept in groups of 10 individuals. The experiment (including the daily administration of PE for the Groups 2 and 3) were then continued for 290 days (9½ months). Food ingestion and the general body condition were appreciated weekly throughout the experiment. Rats were examined once a week to evaluate the possible existence and location of mammary tumors.

Due to welfare grounds and destabilizing body condition induced by the tumor progression in some individuals, the experiment was stopped at 290 days from MNU-inoculation (or after 294 days of daily intake of PE). At the end of the study, animals from all four groups were humanly exsanguinated after deep narcosis with halothane. The following details were recorded in all groups: the number of developed tumors, incidence, multiplicity, size of the tumor mass, and weight of the tumor mass. The size of each tumor was assessed using a micrometer caliper.

### 2.4. Necropsy and Histopathology

A complete necropsy survey was performed on the biological material used in the study. Concerning the gross examination of the tumors detected throughout the body, the following details were recorded: the mass location, dimension, weight, consistency, and the status of regional lymph nodes (e.g., hypertrophy, mobility). Additionally, for each rat from, the Groups 1 and 2 the subsequent parameters of the detected mammary tumors were calculated: (a) the percentage of the mammary tumor mass (MTm) relative to the final body weight (FBW) of the rat, by using the formula: MTm(g) × 100/FBW(g) = MTm(%); (b) the volume of each tumor was calculated using the formula suggested by Woditschka et al. (2008): 0.5 × tumor length × tumor width × tumor height [31]; (c) mammary tumors multiplicity (MTM) for the Groups 1 and 2 (i.e., average mammary tumor number/rat in each of the two groups).

Furthermore, necropsy included a gross and histological examination of the internal organs. In all animals, several tissue samples were harvested from a number of organs (e.g., stomach, intestine, pancreas, liver, lung, spleen, kidney, lymph nodes, and central nervous system) for further histological inspection. Harvested samples were fixed in 10% buffered formalin, embedded later in paraffin blocks, and eventually, the tissue sections (5 *μ*m in thickness) were stained by hematoxylin and eosin procedure. The mammary tumors were categorized according to their histological type and as benign or malignant [32]. In the case of malignant mammary tumors, a grading system was utilized [33].

### 2.5. Assays of Serum Components and Enzymes

Serum analysis of various liver marker enzymes such as total protein, albumin, globulin, alkaline phosphatase (ALP), alanine aminotransferase (ALT), amylase (AMY), calcium (Ca), phosphorus (Pa), sodium (Na), potassium (K), blood urea nitrogen (BUN), total bilirubin (TBIL), creatinine (CRE), and glucose (GLU) were estimated by using VetScan® Comprehensive Diagnostic Profile reagent rotor used with the VetScan Chemistry Analyzer (Diamond Diagnostics, Holliston, MA, USA).

### 2.6. Assays of Antioxidant Profile

#### 2.6.1. Total Protein Extract

Samples of hepatic tissue were twice washed with saline solution. Then each sample of hepatic tissue was homogenized using an ultraturax and the total proteins were extracted with potassium phosphate buffer (50 mM, pH 7.35) [34]. Samples were analyzed by determining the total proteins concentration, the activity of antioxidant enzymes (SOD, CAT, GPx), the lipid peroxidation level, and the amount of oxidized proteins produced in the liver of MNU- and/or PE-exposed rats as previously was assessed in our laboratory [35].

#### 2.6.2. Superoxide Dismutase Assay

Superoxide dismutase (SOD) assay kit (Cayman Chemical Company, Michigan, USA) is based on the conversion of a tetrazolium salt to formazan by superoxide radicals generated in xanthine/xanthinoxidase system. The current method measures the activities of all three SOD types. One unit of enzyme is defined as the amount of enzyme needed to exhibit 50% dismutation of the superoxide radical. Absorbances were monitored at 450 nm by HT BioTek Synergy (BioTek Instruments, USA) microplate plate reader.

#### 2.6.3. Catalase Assay

Catalase (CAT) assay kit (Cayman Chemical Company, Michigan, USA) is based on the reaction of the enzyme with methanol in the presence of H_2_O_2_. The formaldehyde produced is measured colorimetrically with 4-amino-3-hydrazino-5-mercapto-1,2,4-triazole (Purpald) as the chromogen. A standard curve of bovine liver catalase was used for the determination of enzyme activity. One unit is defined as the amount of enzyme that will cause the formation of 1 nmol of formaldehyde per minute at 25°C. Absorbances were recorded at 540 nm wavelength by using microplate plate reader HT BioTek Synergy (BioTek Instruments, USA).

#### 2.6.4. Glutathione Peroxidase Assay

Glutathione peroxidase activity (GPx) Ransel kit (Randox Laboratories Ltd., London, UK) is based on the oxidation reaction of glutathione (GSH), using cumene hydroperoxide as a substrate. In the presence of glutathione reductase (GR) and NADPH+H+, glutathione disulfide (GSSG) is reduced to sulfhydryl form glutathione (GSH). This mechanism is possible due to the oxidation process of NADPH into NADP+. The decrease in absorbance was measured at 340 nm wavelength by using microplate plate reader HT BioTek Synergy (BioTek Instruments, USA).

#### 2.6.5. Determination of Lipids Peroxidation Level

The most common indicator of peroxidation is the chemical compound malondialdehyde (MDA), being the final degradation product resulted in lipids peroxidation. The assay is based on the interaction between the resulted MDA and thiobarbituric acid (TBA). The MDA-TBA adduct formed by the reaction between the resulted MDA and thiobarbituric acid (TBA) under high temperature (90–100°C) and acidic conditions was measured colorimetrically at 540 nm, by using microplate plate reader HT BioTek Synergy (BioTek Instruments, USA). The protocol assay TBARS kit was followed as the producer (Cayman Chemical Company, Michigan, USA) indicated.

#### 2.6.6. Determination of Protein Degree Oxidation

The most significant two compounds resulted from the oxidation process of proteins are glutamic semialdehyde and aminoadipic semialdehyde. The method recommended by the assay kit (Cayman Chemical Company, Michigan, USA) is based on the reaction between aldehydes and 2,4 dinitrophenylhydrazine (DNPH). Products resulted from protein oxidation processes reacted with DNPH and formed a colorful compound, which was quantified spectrophotometrically. The quantity of DNPH that reacted with the products obtained due to the oxidation process was expressed as g protein per g tissue.

## 3. Results

### 3.1. Characterization of Propolis Extract (PE)

The presence of phenolic compounds in PE was verified by the Folin-Ciocalteu reaction and total phenolic content was 46.27 ± 1.18. Moreover, the PE was subjected to the spectrophotometric analysis for total flavones and flavonols content assessment by using the method based on aluminum chloride complex formation and the value obtained was 7.90 ± 0.32%. Using the colorimetric method with dinitrophenylhydrazine, the total flavanone and dihydroflavonol content of PE was 3.85 ± 0.61%.

### 3.2. Chemopreventive Effects of Propolis on Mammary Tumor Development

**Figure 1:**
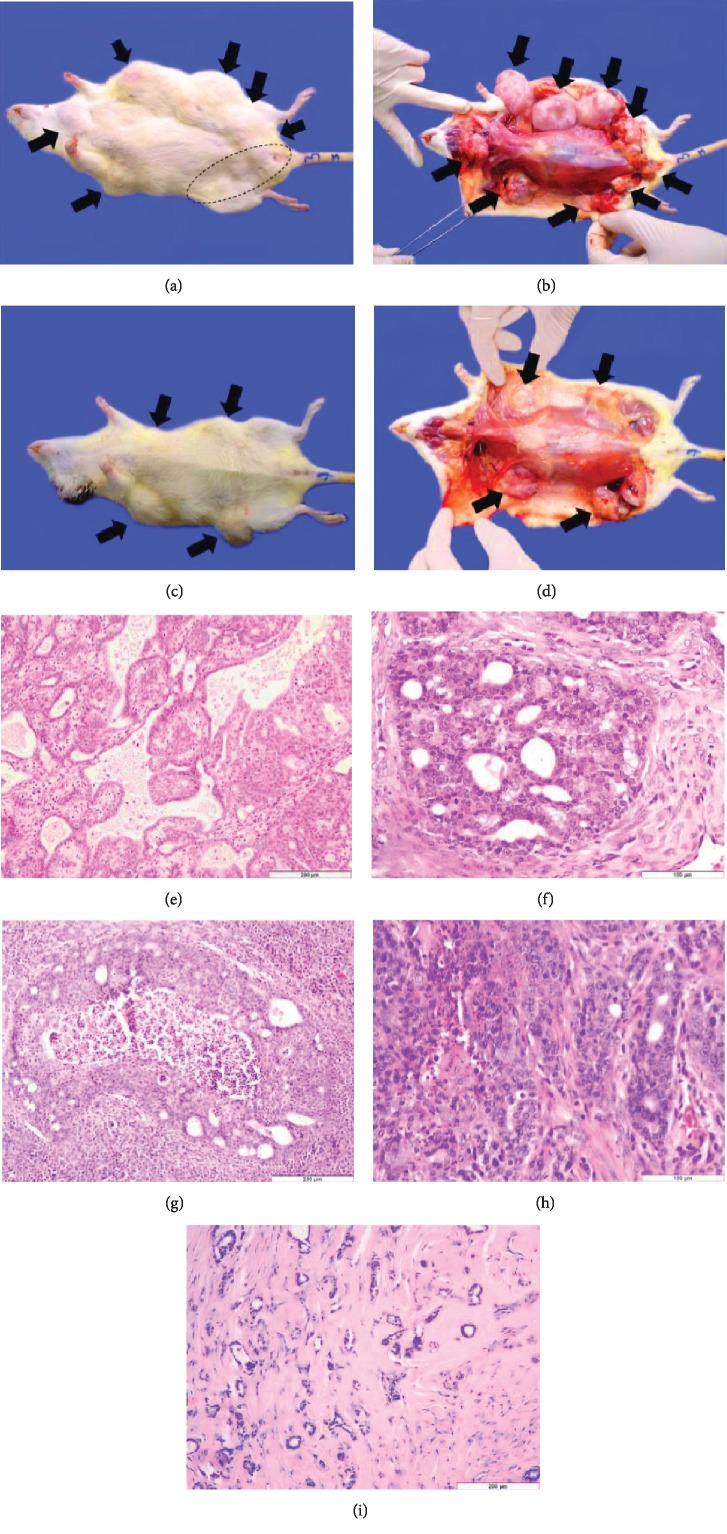
(a) MNU-induced mammary tumors visible as subcutaneous masses of different sizes (arrows and selected area); Rat no. 3, Group 1. (b) Gross features of MNU-induced mammary tumors following skinning (arrows); Rat no. 3, Group 1. (c) and (d) are comparative features regarding the location and gross features of the MNU-induced mammary tumors (arrows) in Group 2 individuals (Rat no. 7). (e) Invasive tubular carcinoma, grade 1, represented by tubular and tubulopapillar structures; HE stain (Group 1, Rat 1, 5^th^ left mammary gland). (f) Ductal *in situ* cribriform carcinoma, grade 2, made of a solid proliferation with formation of secondary lumina; HE stain (Group 1, Rat 3, 1^st^ right mammary gland). (g) Ductal *in situ* comedo-carcinoma, grade 2, which appears as distended ductal structures with a multilayered epithelium surrounding a necrotized area; HE stain (Group 1, 2^nd^ left mammary gland). (h) Invasive tubular carcinoma, grade 3, composed of tubular structures with increased nuclear size and prominent nucleoli; HE stain (Group 2, Rat 4, 2^nd^ left mammary gland). (i) Fibroadenoma composed of ductal structures surrounded by fibrous tissue; HE stain (Group 2, Rat 9, 4^th^ right mammary gland).

The tumor development occurred only in subjects of Groups 1 and 2, i.e., MNU-exposed rats. In Group 1, all animals developed tumors (Figures 1(a) and 1(b)), in contrast to rats of Group 2 where only 7 out of 10 presented tumors (Figures 1(c) and 1(d)). It was observed in Group 1 a variation of 1 to 9 tumors/rat; meanwhile in Group 2, 0 up to 5 tumors/rat occurred (Tables 1 and 2).

Microscopically, mammary tumors were classified as benign or malignant (in situ or invasive) mammary carcinomas. In the case of malignant mammary lesions, in Group 1 were identified 64.86% of grade I (Figure 1(e)), 18.91% of grade II, and 2.7% of grade III mammary carcinomas (Figures 1(f) and 1(g)), the rest of 13.51% being benign mammary lesions. In the case of Group 2, the abundance of identified mammary carcinomas of grade I was 38.88%, of grade II was 33.33%, and 16.66% of grade III (Figure 1(h)), while 11.11% were benign mammary lesions (Figure 1(i)). Histologically, mammary tumor types detected did not present structural differences in subjects from Group 1 versus Group 2.

The total number of tumors recorded in the Group 1 was 41 (i.e., 37 mammary and 4 non-mammary tumors), whereas subjects of Group 2 developed 20 tumors (i.e., 18 mammary and 2 non-mammary tumors). Therefore, the mammary tumors induction ratio was 90.24% in Group 1, respective to 90% in Group 2. Regarding the mammary tumors multiplicity, a significant variation occurred between both groups as follows: 3.7 ± 2.75 mammary tumors/rat in Group 1 and 1.8 ± 1.39 mammary tumors/rat in Group 2 (*p* < 0.001) (Table 3). Moreover, in Table 3 can be seen the difference in the average of mammary tumors volume (16.68 ± 33.32 in Group 1 vs 10.76 ± 17.17 in Group 2; *p* > 0.05) and average MTM (%) relative to FBW (9.00 ± 10.86 in Group 1 vs 7.42 ± 11.43 in Group 2; *p* > 0.05).

### 3.3. Blood Biochemical Profile in All Experimental Groups

Blood biochemical parameters of rats inoculated with normal saline solution were taken as reference values. In general, inoculation of MNU (Group 1) caused a slight decrement of total blood proteins, associated with a significant decrease of albumins and a very significant increase of globulins. Concerning the activity of sanguine enzymes, ALT was slightly increased, but ALP and AMY were non-significantly decreased in MNU-inoculated rats comparing with control (Group 4) (Table 4). The rats MNU-treated had levels of TBIL, BUN, and CRE decreased in contrast to rats of control Group 4. The concentration of GLU in blood of MNU-inoculated rats assessed was highly significant than that of rats from the control group. On the other hand, levels of microminerals were non-statistically changed after the MNU-inoculation.

The administration of PE into the diet of MNU-inoculated rats did not change total proteins concentration but slightly increased albumins and significantly decreased globulins. The activity of ALT enzyme decreased and AMY increased, but non-statistically relevant, in MNU-inoculated/PE-treated rats comparing with MNU-inoculated ones (Group 1). Hematological values of BUN, GLU (statistic relevant, *p* < 0.0001) for MNU-inoculated/PE-treated rats were lower than of MNU-inoculated rats, but increased levels of TBIL and CRE were recorded. Microminerals profile was not statistically significant modified by the PE administration in the diet of MNU-inoculated rats. Overall, varying strengths of PE in the diet of MNU-inoculated rats showed a potentiating effect on albumins, AMY, TBIL, and CRE while a lowering effect on globulins, ALT, BUN, and GLU compared with that of MNU-treated rats. PE from the diet of MNU-inoculated rats seems able to restore these values close to the physiological normal ones.

### 3.4. The Effects of MNU and Propolis on the Antioxidative Status

**Figure 2:**
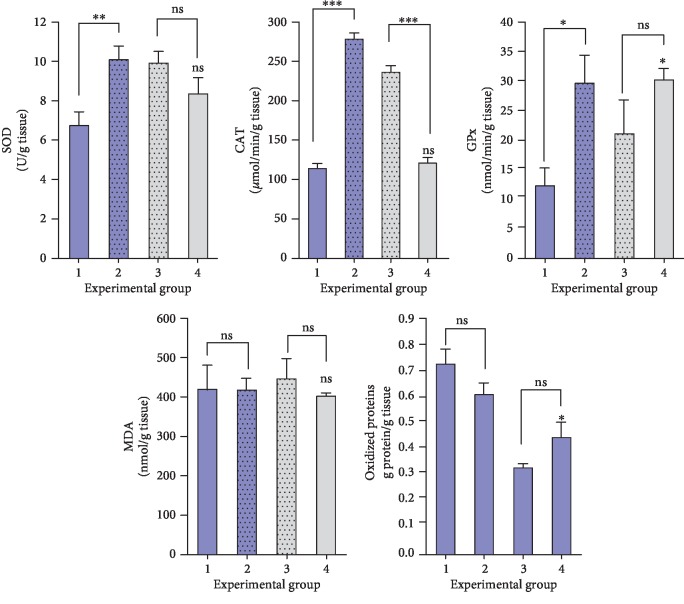
Effect of MNU-inoculation and propolis diet administration on hepatic antioxidative markers in rats. Statistically, all groups were compared with Group 1 (MNU-inoculated), respective to Group 4 control (normal saline inoculation and normal rat food) (^∗^*p* < 0.05, ^∗∗^*p* < 0.01, ^∗∗∗^*p* < 0.0001). Comparisons for the antioxidant profile were made on the basis of the one-way ANOVA followed by Bonferroni's test (GraphPad Prism version 6.07).

Inoculation of the MNU carcinogenic agent produced a significant drop of antioxidant enzymes (SOD, CAT, GPx) in rats, in contrast to control (Figure 2). The PE administration in the diet of rats determined substantial increases of all three antioxidant enzymes levels. Moreover PE induced a significant decrease of the oxidized proteins, increased previously by the MNU-inoculation (Figure 2). The PE presence in the diet of MNU-inoculated rats did not induce significant changes in levels of malondialdehyde (MDA) (Figure 2). The oxidized proteins level in MNU-inoculated rats increased in contrast to rats of the group considered control. It seems that after the PE administration in the diet of MNU-inoculated rats, the level of oxidized proteins is decreased, but data obtained are not statistically relevant (Figure 2).

## 4. Discussion

Breast cancer is the leading cause of cancer-related deaths among women worldwide. Literature studies sustain that the breast cancer, a heterogeneous tumor, has a varying response to treatments. Despite the improved efficacy offered by modern treatments, their toxicity and often unpleasant side-effects remain a major source of concern for patients and clinicians. New approaches to improve tolerance of the cancer chemotherapy are urgently needed and the present topic focuses on this issue. Over the time, the biological properties of propolis collected from different areas of the world to exert antiproliferative and cytotoxic potential toward tumor cells were evaluated and observed [10, 13, 16, 20, 36]. A recent study sustains that the biological activity of propolis is positively influenced by the presence of flavonoids and phenolic acids in it [29]. Therefore for this study, a propolis sample collected from Transylvania, rich in phenolic acids 46.27 ± 1.18%, containing flavones/flavonols (7.90 ± 0.32%) and flavanone 3.85 ± 0.61% was selected. The biochemical characterization reported here for the Transylvanian propolis is in accordance with that reported in literature for a typical poplar propolis [37].

Our study highlights some data regarding the propolis usage as a potential chemopreventive agent.

Sprague–Dawley rat model was selected to be the animal model for the current study, because it bears histological features of mammary tumors closely to that of human and canine mammary tumors. As an agent to induce mammary adenocarcinomas in female rats, the N-methyl-N-nitrosourea (MNU) agent was used to be inoculated intra peritoneal to healthy rats, because the mammary tumors induced by it seem to be more aggressive and their occurrence higher [38, 39]. Moreover, MNU-induced rat mammary tumors possess some similar features of human breast cancer, such as the growth addiction on ovarian hormones [40] and presence of lymphocytic infiltrates in the reactive stroma [38]. In our study, the mammary tumors induction ratio by MNU was around 90% in both Groups 1 and 2. Histological analysis of mammary tumor sections classified tumors as benign or malignant (*in situ* or invasive) mammary carcinomas. No histomorphological differences were related to the chemoprevention using propolis.

Results of our study demonstrate that a diet containing PE is able to slow down the progression of breast tumors development with lower multiplicity, weight, and size compared with control. 30% of rats from Group 2, MNU-induced/PE-treated, were found to have no tumor at the end of the study. Moreover, the number of grade I tumors recorded for MNU-inoculated rats was 24 and decreased to 7 tumors after the PE administration in the diet of rats MNU-inoculated. Our findings are consistent with another previous in vivo study investigated by Ahmed and colleagues that another bee product as honey may modulate tumor latency, incidence, multiplicity, and progression [41].

It has been demonstrated that tumors can be eliminated or diminished by chronic administration of low doses of chemotherapeutic drugs [42]. It is quite possible that propolis treatment behaves similarly, because doses used in the current study are about 1 mg/body weight/day. As a well-known natural product, being widely consumed by humans, propolis seems to be associated with a high rate of allergic reactions rather than toxicity. It was demonstrated that no toxicity effect was observed for a propolis dose of 1400 mg/body weight/day administered to each mouse in a study with 90 animals.

Regarding the sanguine biochemical profile, the administration of MNU-inoculation in Group 1 of rats triggered a negligible reduction of total blood proteins, a decrease of albumins concentration, and an increase of globulins. Hypoalbuminemia observed in MNU-exposed group can be a consequence of the injurious effects of the MNU carcinogen on the hepatic polyribosomes and protein-soluble factors, and the result of alkylation and carboxylation of RNA and proteins [43]. However, the negligible reduction of total blood proteins can be a consequence of proteolysis. The hydrolytic degradation of proteins can be done directly, by protein oxidation, or indirectly by their increased susceptibility to proteolytic enzymes [44].

In the current study, the MNU-exposed group triggered a major increase in blood glucose, but its effect on blood minerals was insignificant. The insignificant activity of sanguine enzymes (i.e., ALP, ALT, and AMY) was observed in rats MNU-inoculated comparing with those recorded for control group.

PE included in the diet of MNU-inoculated rats seems able to restore these values close to the physiological normal ones. Overall, varying strengths of PE in the diet of MNU-inoculated rats showed a tendency to restore the concentration of albumins and globulins, and to normalize the biochemical blood parameters TBIL, CRE, and GLU values and the activity of ALT enzyme. There is evidence that propolis is able to normalize ALT activity by different doses of propolis in female albino rats of Sprague–Dawley strain [45]. Propolis administered to rats, previously aluminum chloride–treated, normalized the increased transaminases and lactate dehydrogenase (LDH) activity [46].

The enzymatic antioxidant system is composed of superoxide dismutase (SOD), catalase (CAT), and glutathione peroxidase (GPx), which scavenges the reactive oxygen species and lipid peroxidation. It is known that tumor cells exhibit heterogeneity in the levels of oxidative stress and for this reason could have various levels of antioxidant enzymes. Several reports have cited decreased activities of SOD and catalase in various carcinogenic conditions [47–49]. SOD is able to disrupt the potent oxidizing radicals such as superoxide radicals, which are highly diffusible and thus being able to pass through cell membranes causing injuries far apart the tumor site [50]. The superoxide radicals are converted by SOD to hydrogen peroxide and oxygen. CAT is able to catalyse the breakdown of hydrogen peroxide produced by tumor cells. Another equally important antioxidant enzyme involved in the preventing intracellular damage caused by hydrogen peroxide is GPx. In tumor cells, low activities of GPx were reported, maybe due to the altered antioxidant defense system caused by enormous production of free radicals in DMBA-induced carcinogenesis [47].

In the present study, the activity of antioxidant enzymes determined from the liver of exposed rats to MNU carcinogen agent was decreased significantly from a statistical point of view if comparing with control rats, exposed only to normal saline solution. The PE administration in the diet of rats previously MNU-inoculated determined an increment of the antioxidative enzymes (SOD, CAT, GPx) analyzed. A similar effect was reported for propolis and paclitaxel treatment, which increased the activities of enzymatic antioxidants SOD, CAT, and GPx in rats-treated compared with breast cancer-bearing animals treated with either paclitaxel or propolis alone [51].

Malondialdehyde (MDA) is an indicator of lipid peroxidation, which was found to be increased in various cancers, including breast cancer. In the lipid peroxidation process, the polyunsaturated fatty acids (PUFA) in cell membranes are oxidized by reactive oxygen species, resulting metabolites such as MDA, 4-hydroxynoneal (4-HNF) and acrolein. These metabolites bind to proteins and induce functional changes, then cause enzyme inhibition and receptor changes and consequently cell injury [52]. In our study, no significant modification in MDA level was recorded for MNU-inoculated rats or MNU-inoculated/PE-treated rats as compared with control group. In a recent study, da Silvera et al. (2016) observed that the ethanolic extract of yellow propolis induced a decrease in the production of malondialdehyde and nitric oxide in 3-months-old Wistar rats, without affecting levels of superoxide dismutase and catalase antioxidative enzymes [53].

Protein carbonyl levels are the most frequently used biomarker of protein oxidation, because increased levels may be associated with breast cancer risk [54]. Carbonyl groups are formed during the oxidation of protein side chains, mainly on proline, arginine, lysine, and threonine residues [55].

In our study, the oxidized proteins level in MNU-inoculated rats increased. It seems that the PE administration in the diet of MNU-inoculated rats could decrease the level of oxidized proteins, but our data are not statistically relevant. Literature data come to support our evidence and attest that propolis supplementation leads to a reduction in protein oxidation, together with a lowering effect on the level of glucose and cholesterol [56].

The biological effects exhibited by Transylvanian propolis could be related to an overall effect of the phenolic compounds present in the extract. For instance, CAPE (caffeic acid phenethyl ester) is known to be one of the major biologically active principles in propolis with chemoprevention and antitumor properties [18]. CAPE is able to inhibit the nuclear factor kappa B (NF-*κ*B) and to induce apoptosis in breast cancer cells [57]. In tumor cells, transformation via NF-*κ*B results also elevated ROS levels, accompanied with downregulation of cellular antioxidant enzyme systems [58]. Inhibition of antioxidant enzymes is considered a therapeutic approach in the induction of ROS production in tumor cells. Recently, it was proved that propolis can upregulate the intracellular ROS, decrease mitochondrial membrane potential, and induce apoptosis in MCF-7 and MDA-MB-231 cells [11].

## 5. Conclusions

Taken together, these data indicate that propolis could be a chemopreventive agent against MNU-induced mammary carcinogenesis. However, before declaring propolis a chemopreventive agent against human breast cancer, further investigations are needed for a complete identification and characterization of specific bioactive molecules with biological properties and for finding its proper mechanism of action at mammary gland level.

## Figures and Tables

**Table 1:** Mammary tumor occurrence in Group 1: histological features, tumors location, and size; non-mammary tumors detected.

Rat no.	Group 1 (MNU)			
	Mammary tumor type	Mammary tumor size (cm)	MTM(%)^1^ relative to FBW^2^	Other tumor types
1	Invasive tubular carcinoma, G-1 (M_1_ right)	1.3/1.3	17.39	—
	Carcinosarcoma, G-1 (M_4_ right)	3.5/2.6		
	Invasive tubular carcinoma, G-1 (M_2_ left)	1.3/2.1		
	Invasive tubular carcinoma, G-1 (M_5_ left)	6.8/3.5		

2	Invasive cribriform carcinoma, G-1 (M_1_ right)	1.4/1	10.78	Interstitial renal tumor (1.3/1 cm)
	Ductal *in situ* cribriform carcinoma, G-1 (M_2_ right)	1.7/1.2		
	Invasive tubular carcinoma, G-2 (M_3_ right)	6.0/3.5		
	Invasive tubular carcinoma, G-1 (M_5_ right)	1.7/1.2		
	Ductal *in situ* cribriform carcinoma, G-2 (M_2_ left)	2.4/2.2		
	Invasive tubular carcinoma, G-1 (M_3_ left)	2.1/2.0		
	Invasive tubular carcinoma, G-1 (M_5_ left)	1.3/0.9		

3	Ductal *in situ* cribriform carcinoma, G-1 (M_1_ right)	3/2.5	29.27	—
	Invasive tubular carcinoma, G-1 (M_3_ right)	3.5/3		
	Ductal *in situ* solid carcinoma, G-1 (M_4_ right)	2.5/2.2		
	Invasive tubular carcinoma, G-1 (M_5_ right)	1.3/1.2		
	Invasive tubular carcinoma, G-1 (M_1_ left)	2.5/2		
	Fibroadenoma (M_2_ left)	5/4.5		
	Ductal *in situ* papillary carcinoma, G-1 (M_3_ left)	6.5/5.1		
	Invasive tubular carcinoma, G-1 (M_4_ left)	6.2/5.6		
	Ductal *in situ* papillary carcinoma, G-1 (M_5_ left)	4/3.5		

4	Ductal *in situ* solid carcinoma, G-3 (M_2_ right)	0.8/0.5	23.87	Malignant lymphoma (diffuse in both mammary chains)
	Ductal *in situ* cribriform carcinoma, G-1 (M_5_ right)	7.9/6.5		
	Ductal *in situ* comedo-carcinoma, G-2 (M_2_ left)	3/2.7		
	Ductal *in situ* papillary carcinoma, G-1 (M_4_ left)	5.4/3.2		

5	Fibroadenoma (M_4_ right)	1.7/1.4	0.25	—

6	Ductal *in situ* cribriform carcinoma, G-2 (M_1_ right)	0.8/0.6	4.76	—
	Invasive tubular carcinoma, G-2 (M_3_ left)	4.2/3.7		

7	Invasive tubular carcinoma, G-1 (M_1_ right)	1.2/0.5	0.97	—
	Adenoma (M_2_ right)	1.1/1		
	Adenoma (M_2_ left)	1.0/0.7		
	Invasive papillary carcinoma, G-1 (M_3_ left)	0.9/0.9		
	Invasive tubular carcinoma, G-1 (M_4_ left)	0.9/0.6		

8	Invasive tubular carcinoma, G-2 (M_2_ right)	1.3/1.2	1.82	—
	Invasive tubular carcinoma, G-1 (M_2_ left)	2.8/2.3		

9	—	—	—	Liposarcoma in the omentum (1.7/1.2 cm)
				Ovarian fibrosarcoma (1.5/1.2 cm)

10	Invasive tubular carcinoma, G-2 (M_3_ right)	1.2/1.2	0.95	—
	Fibroadenoma (M_1_ left)	1.4/1.2		
	Ductal in situ cribriform carcinoma, G-1 (M_5_ left)	1.2/1		

^1^MTm: mammary tumor mass; ^2^FBW: final body weight; MNU: N-methyl-N-nitrosourea; M_1-5_: mammary gland number and its side (i.e., right, left); G: histological grade.

**Table 2:** Chemopreventive effects of propolis on mammary tumor occurrence in Group 2: histological features, tumors location, and size; non-mammary tumors detected.

Rat no.	Group 2 (MNU+PE)			
	Mammary tumor type	Mammary tumor size (cm)	MTM(%)^1^ relative to FBW^2^	Other tumor types
1	Invasive cribriform carcinoma, G-1 (M_1_ left)	1.5/1.5	22	—
	Invasive cribriform carcinoma, G-3 (M_2_ left)	4.5/3		
	Invasive cribriform carcinoma, G-2 (M_5_ left)	7.5/4.4		

2	Invasive tubular carcinoma, G-2 (M_4_ left)	3.5/2.4	32.8	Rhabdomyosarcoma (diaphragm)
	Invasive tubular carcinoma, G-2 (M_5_ left)	4.5/2.2		
	Invasive tubular carcinoma, G-1 (M_1_ right)	1.5/0.8		

3	Invasive tubular carcinoma, G-2 (M_1_ right)	2.5/2.8	1.33	
	Invasive papillary carcinoma, G-2 (M_1_ left)	1.1/0.6		

4	Invasive tubular carcinoma, G-3 (M_1_ left)	1/0.7	5.78	—
	Invasive tubular carcinoma, G-3 (M_2_ left)	5.5/3.2		

5	—	—	—	—

6	—	—	—	—

7	Invasive tubular carcinoma, G-1 (M_2_ right)	3.5/3.2	11.56	Squamous carcinoma (facial skin)
	Invasive tubular carcinoma, G-2 (M_4_ right)	5/3.5		
	Invasive tubular carcinoma, G-1 (M_2_ left)	3.5/1.5		
	Invasive tubular carcinoma, G-1 (M_4_ left)	5.2/2.5		

8	Ductal *in situ* cribriform carcinoma, G-1 (M_4_ right)	1.1/0.6	0.55	
	Invasive tubular carcinoma, G-1 (M_2_ left)	1.8/1.3		

9	Fibroadenoma (M_4_ right)	1.2/1.1	0.22	—
	Fibroadenoma (M_1_ left)	1.1/1		

10	—	—	—	—

^1^MTm: mammary tumor mass; ^2^FBW: final body weight; MNU: N-methyl-N-nitrosourea; PE: propolis; M_1-5_: mammary gland number and its side (i.e., right, left); G: histological grade.

**Table 3:** Comparative data concerning mammary and non-mammary tumors developed in rats of Groups 1 and 2.

Experimental group	Multiplicity^1^	Average MTM(%)^2^ relative to FBW^3^	Average mammary tumors volume^4^	Total number of mammary tumors/group	Non-mammary tumors/group
Group 1	3.7 ± 2.75	9.00 ± 10.86	16.68 ± 33.32	37	4
Group 2	1.8 ± 1.39^ns^	7.42 ± 11.43^ns^	10.76 ± 17.17^ns^	18	2

^1^Multiplicity (i.e., average mammary tumor number/rat); ^2^MTM: mammary tumor mass; ^3^FBW: final body weight; ^4^Mammary tumor volume calculated using the formula suggested by Woditschka et al., 2008. In order to determine significant differences between mean values, Student's *t* test was used (GraphPad Prism version 6.07).

**Table 4:** Modulatory influences of MNU and propolis on blood biochemical parameters.

Blood parameter	Experimental group			
	Group 1 MNU	Group 2 MNU/PE	Group 3 PE	Group 4 Control
Blood proteins				
Total proteins (g/dL)	7.08 ± 0.77^ns^	7.05 ± 1.34^ns^	7.37 ± 0.17^ns^	7.66 ± 0.44
Albumins (g/dL)	4.98 ± 1.07^∗^	5.45 ± 1.31^ns^	5.35 ± 0.29^ns^	6.06 ± 0.24
Globulins (g/dL)	2.08±0.43^∗∗^	1.70 ± 0.22^#^	2.15±0.26^∗∗∗^	1.60 ± 0.21
The activity of blood enzymes				
ALP (U/L)	147.71 ± 72.24^ns^	147.71 ± 72.24^ns^	130.00 ± 3.16^ns^	151.66 ± 53.09
ALT (U/L)	117.14 ± 85.40^ns^	80.66 ± 35.53^ns^	70.50 ± 1.50^ns^	98.33 ± 31.04
AMY (U/L)	527.28 ± 96.89^ns^	628.16 ± 112.82^ns^	615.75 ± 166.09^ns^	592.66 ± 29.10
Blood biochemical parameters				
TBIL (mg/dL)	0.21 ± 0.09^∗^	0.22 ± 0.04^ns^	0.33 ± 0.04^ns^	0.30 ± 0.08
BUN (mg/dL)	16.00 ± 2.00^ns^	14.83 ± 2.60^ns^	12.75±1.08^∗∗∗^	17.66 ± 1.24
CRE (mg/dL)	0.18±0.09^∗∗^	0.20 ± 0.05^ns^	0.22 ± 0.08^∗^	0.30 ± 0.06
GLU (mg/dL)	158.25±5.26^∗∗∗^	97.33 ± 26.78^###^	48.83 ± 16.72^ns^	61.00 ± 13.20
Blood microminerals				
Ca (mg/dL)	11.77 ± 1.09^ns^	11.11 ± 2.26^ns^	11.37 ± 0.28^ns^	11.53 ± 0.23
Pa (mg/dL)	6.48 ± 1.86^ns^	5.71 ± 1.54^ns^	5.10 ± 0.15^ns^	5.30 ± 0.28
Na (mmol/L)	144.14±1.24^∗∗^	142.60 ± 1.85^ns^	142.25 ± 1.47^ns^	142.00 ± 1.41
K (mmol/L)	8.50 ± 0.49^ns^	8.00 ± 1.4^ns^	7.70 ± 0.78^ns^	7.60 ± 0.21^ns^

Values are mean ± SD. Each group contains ten animals. Comparisons were made on the basis of the one-way ANOVA followed by Dunnett's test (GraphPad Prism version 6.07). Group 1 (MNU-inoculated rats) and Group 3 (PE-treated rats) were compared with the normal control group (Group 4) (ns: nonsignificant, ^∗^*p* < 0.05, ^∗∗^*p* < 0.01, ^∗∗∗^*p* < 0.0001), respective Group 1 MNU-inoculated with Group 2 (MNU-inoculated/PE-treated) (MNU) (ns: nonsignificant, ^#^*p* < 0.05, ^##^*p* < 0.01, ^###^*p* < 0.0001).

## Data Availability

The data used to support the findings of this study are available from the corresponding author Rugina D. upon request (email: dumitrita.rugina@usamvcluj.ro).

## Supplementary Materials

Supplementary Materials Propolis reduced the number of grade I tumors in MNU-induced/propolis-treated rats. 4 propolis increased the levels of antioxidative enzymes (SOD, CAT, and GPx) and restored blood 5 parameters values close to the physiological normal ones.
