# Supplementary figures and images for: Chemopreventive Effects of Propolis in the MNU-Induced Rat Mammary Tumor Model
Source: Oxid Med Cell Longev. 2020 Feb 26;2020:4014838. doi: 10.1155/2020/4014838 (PMC7063188; doi:10.1155/2020/4014838)

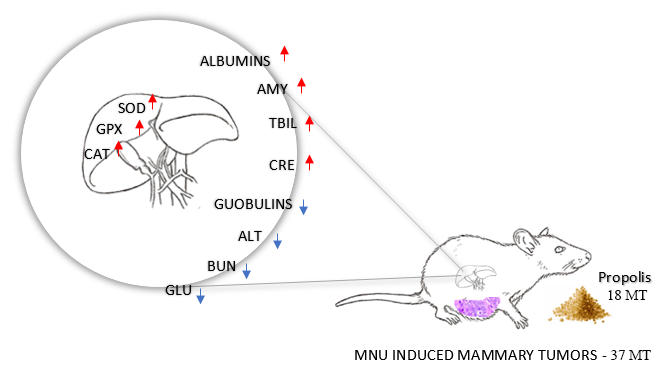

Supplement: Supplementary Materials — Propolis reduced the number of grade I tumors in MNU-induced/propolis-treated rats. 4 propolis increased the levels of antioxidative enzymes (SOD, CAT, and GPx) and restored blood 5 parameters values close to the physiological normal ones. [file 4014838.f1.tif]
